# Clinical and laboratory characteristics of children with sickle cell disease on hydroxyurea treated with artemether-lumefantrine for acute uncomplicated malaria

**DOI:** 10.3389/fmed.2023.1291330

**Published:** 2023-11-23

**Authors:** Catherine Segbefia, Seth Kwabena Amponsah, Adwoa K. A. Afrane, Mame Yaa Nyarko, Yvonne Brew, Nihad Salifu, Samuel Yao Ahorhorlu, Abdul Malik Sulley, Lars Hviid, Michael Fokuo Ofori, George Obeng Adjei

**Affiliations:** ^1^Department of Child Health, University of Ghana Medical School, Accra, Ghana; ^2^Department of Medical Pharmacology, University of Ghana Medical School, Accra, Ghana; ^3^Princess Marie Louise Hospital, Accra, Ghana; ^4^Department of Paediatrics, Greater Accra Regional Hospital, Accra, Ghana; ^5^Centre for Tropical Clinical Pharmacology and Therapeutics, University of Ghana Medical School, Accra, Ghana; ^6^Centre for Medical Parasitology, Department of Immunology and Microbiology, Faculty of Health and Medical Sciences, University of Copenhagen, Copenhagen, Denmark; ^7^Department of Infectious Diseases, Rigshospitalet, Copenhagen, Denmark; ^8^Department of Immunology, Noguchi Memorial Institute for Medical Research, University of Ghana, Legon, Ghana

**Keywords:** sickle cell disease, malaria, hydroxyurea, children, artemether-lumefantrine

## Abstract

**Introduction:**

Limited information exists on any interactions between hydroxyurea (HU) and antimalarials in sickle cell disease (SCD). We evaluated changes in clinical and laboratory parameters among children with SCD on HU therapy treated with artemether-lumefantrine (AL) for acute uncomplicated malaria (UM).

**Methods:**

A prospective, non-randomized, pilot study of 127 children with SCD (23, UM; 104, steady state) were recruited from three hospitals in Accra. UM participants were treated with standard doses of AL and followed up, on days 1, 2, 3, 7, 14, and 28. Venous blood was collected at baseline and follow-up days in participants with UM for determination of malaria parasitaemia, full blood count, reticulocytes, and clinical chemistry. Further, *Plasmodium falciparum* identification of rapid diagnostic test (RDT) positive samples was done using nested polymerase chain reaction (PCR).

**Results:**

Among SCD participants with UM, admission temperature, neutrophils, alanine-aminotransferase, gamma-glutamyl-transferase, and haemoglobin significantly differed between HU recipients (HU+) and steady state, while white blood cell, neutrophils, reticulocytes, bilirubin, urea, and temperature differed significantly between non-HU recipients (no-HU), and steady state. Mean parasitaemia (HU+, 2930.3 vs. no-HU, 1,060, *p* = 0.74) and adverse events (HU+, 13.9% vs. no-HU, 14.3%), were comparable (*p* = 0.94). Day 28 reticulocyte count was higher in the HU+ (0.24) (0.17 to 0.37) vs. no-HU, [0.15 (0.09 to 0.27), *p* = 0.022]. Significant differences in lymphocyte [HU+ 2.74 95% CI (−5.38 to 58.57) vs. no-HU −0.34 (−3.19 to 4.44), *p* = 0.024]; bilirubin [HU+, −4.44 (−16.36 to 20.74) vs. no-HU −18.37 (−108.79 to −7.16)]; and alanine aminotransferase, [HU+, −4.00 (−48.55 to 6.00) vs. no-HU, 7.00 (−22.00 to 22.00)] were observed during follow up.

**Conclusion:**

Parasite clearance and adverse event occurrence were comparable between SCD children treated with AL irrespective of HU status. However, distinct patterns of changes in laboratory indices suggest the need for larger, more focused studies.

## Introduction

The highest burden of sickle cell disease (SCD) is in sub-Saharan Africa where over 300,000 children are born each year with SCD, and related childhood mortality rates remain high (1, 2). Malaria and SCD have a similar geographic distribution across endemic regions in sub-Saharan Africa and Asia, where malaria is often a major cause of infection-related hospitalization in SCD (3–5). In people with SCD, malaria can worsen baseline anaemia, trigger acute complications of SCD, and increase the likelihood of gram-negative bacteraemia—all of which potentially increase morbidity and risk of death (6, 7).

Hydroxyurea (HU) was the first drug approved as a disease modifying agent in sickle cell anaemia and is recommended for use, irrespective of phenotypic severity. HU has multiple benefits in SCD including increases in absolute haemoglobin (Hb), mean corpuscular volume (MCV) and foetal haemoglobin (HbF) levels, reductions in white blood cell (WBC), neutrophil, platelet, and reticulocyte counts, and increased nitric oxide availability. At the cellular level, these effects inhibit sickle polymerization, reduce inflammation, and improve blood rheology (8, 9).

There was earlier conflicting evidence about the benefits or safety of HU on the predisposition to and clinical course of malaria; however, the REACH trial showed a lower incidence of malaria in children on HU at maximum tolerated dose (10). HU-associated reductions in the absolute neutrophil count (ANC) below 3.0 × 10^9^/L significantly decreased malaria incidence within the study population. The NOHARM study also demonstrated that HU was safe in very young children (ages 1.00–3.99 years), with no difference in malaria incidence or severity between children on HU or placebo. NOHARM study participants received malaria chemoprophylaxis, and there were 12 episodes of clinical malaria over a 1 year follow-up period (11).

Our group has previously shown high (>95%) days 28 and 42 cure rates in SCD patients with acute malaria, using recommended antimalarials such as artemisinin-based combination therapies (ACT). These studies were conducted prior to the introduction of HU as a disease modifying agent in Ghana, where 1 in 50 newborns is affected with SCD (12, 13). It is unknown which changes occur in haematological or biochemical parameters of children on HU therapy who are concomitantly treated with ACT. There are no published reports on the interplay, if any, between HU and ACT during the acute phase of malaria, when significant changes in blood cell parameters may occur, or any influence of HU on parasite clearance from peripheral blood. One of HU’s therapeutic effects in SCD is through transient reduction of intracellular HbS concentration within erythrocyte precursors in favour of HbF, while haemoglobinopathies have been demonstrated to influence the activity of artemisinin based antimalarials by altering their accumulation and binding to target molecules within parasitized erythrocytes (14). Additionally, while artemisinin derivatives are well tolerated and typically do not have major haematological side effects, there are reports of delayed haemolytic anaemia and dose-dependent decreases in neutrophil counts (15, 16). These effects could potentially exacerbate HU and malaria-associated alterations in blood cell parameters. Furthermore, a retrospective study from Portugal in returned adult travellers with non-severe falciparum malaria, found that there were statistically significant elevations in liver enzymes, specifically alanine aminotransferase (ALT) and aspartate aminotransferase (AST), in those who were treated with a standard 3 days course of artemether-lumefantrine (AL) compared to a historical cohort of patients treated with a quinine-doxycycline regimen. These liver enzyme elevations were clinically asymptomatic, did not require treatment interruption and resolved without any specific interventions (17). HU has been implicated in both worsening of hepatic dysfunction in patients with SCD and improvements in sickle cell hepatopathy (18, 19).

Studies that evaluate changes in clinical and laboratory parameters in SCD patients on HU during acute malaria would provide information to help guide optimal malaria treatment policy, especially since HU use in Africa has become more widespread and is considered standard of care in SCD. Therefore, our main study objectives were to: (i) evaluate differences, if any, in selected haematological and clinical chemistry parameters between children with SCD on HU therapy treated with artemether-lumefantrine (AL) for uncomplicated malaria, compared to those not on HU therapy, (ii) determine parasite clearance and treatment efficacy and (iii) frequency of occurrence of adverse events (AEs) in both groups during a 28 days follow up period.

## Materials and methods

### Study design and sites

This was a prospective, non-randomized, pilot study conducted at three sites—Department of Child Health, Korle Bu Teaching Hospital, Greater Accra Regional Hospital, and Princess Marie Louise Children’s Hospital, all in Accra, Ghana. Study recruitment was from June 2021 to August 2022. All three institutions are state-funded public health facilities with specialist paediatricians and paediatric sickle cell clinics. Folate supplementation is given routinely to all children with SCD at the study sites. For patients on HU, Hb F levels are not routinely checked due to the prohibitive cost of testing. Malaria chemoprophylaxis is not used, although families are routinely counselled on other preventive methods like insecticide-treated bed nets. National guidelines for uncomplicated malaria recommend treatment with ACT if there is either a positive malaria rapid diagnostic test (RDT) or blood film (microscopy) showing malaria parasites.

### Study population and inclusion criteria

Inclusion criteria were, (i) history of fever within the previous 72 h or axillary temperature ≥37.5°C at the time of presentation to the health facility, (ii) positive malaria RDT, and (iii) decision by attending physician to treat as uncomplicated malaria with ACT. Children with signs and symptoms of severe malaria using WHO criteria (e.g., prostration, seizures, coma, or other neurologic abnormalities etc.) were excluded.

One hundred and twenty-seven children, ages one to 15 years, were prospectively recruited as follows: twenty-three (23) children with SCD and RDT-positive uncomplicated malaria who received AL (10 on HU and 13 not on HU) were enrolled and followed up. Additionally, 104 children with SCD in “steady state” reporting to the study sites for routine quarterly follow up clinic visits (58 on HU and 46 not on HU) were enrolled as comparators for the SCD RDT-positive participants. Steady state SCD was defined as clinically asymptomatic with no pain, fever or other intercurrent illness in the 4 weeks prior to recruitment, no blood transfusions in the preceding 4 months (20), and a negative RDT test on day of enrolment.

### Recruitment and baseline procedures (day 0)

Baseline screening included clinical history, physical examination, malaria RDT (Biozek Medical, Netherlands), and completion of a structured case record form by study physicians. We collected 2 mL of venous blood into ethylenediaminetetraacetic acid (EDTA) and heparinized tubes for determination of malaria parasitaemia, full blood count, reticulocytes (RET) and clinical chemistry, in all participants with a positive RDT test result. RDT-positive individuals were treated with a 3 days course of AL per manufacturer’s weight-based dosing guidelines. AL was supervised by caregivers of patients treated on outpatient department (OPD) basis. For those who were admitted, the nursing staff supervised their AL course.

### Follow-up assessments

A complete physical examination, assessment for new or persistent symptoms, AEs, and malaria parasitaemia were done on follow up days 1, 2, 3, 7, 14, and 28. Full blood count, RET and clinical chemistry were repeated on days 3, 7, 14, and 28. Participants in steady state did not require follow up. AEs were assessed during follow up visits and those of grade two or higher [according to the Common Terminology Criteria for Adverse Events (CTCAE) version 5] were documented for all participants.

### Laboratory procedures

Thick and thin blood smears were prepared, stained with Giemsa, and parasite density was determined by counting the number of asexual stage parasites relative to 200 WBC, multiplied by the measured WBC count. Each slide was read independently by two certified microscopists and the average of two readings recorded. Full blood count with leucocyte differential, absolute neutrophil count (ANC) and RET were done using an automated haematology analyzer (Sysmex XN-350, Japan). Clinical chemistry (gamma-glutamyl-transferase, aspartate aminotransferase, alanine aminotransferase, total bilirubin, serum creatinine, urea, serum potassium, and serum sodium) was done with the HumaLyte Plus 5 and HumaStar 100 chemistry analyzer systems (Germany). *Plasmodium falciparum* identification of RDT positive samples was done by extracting genomic DNA from dried blood spots using a chelex extraction procedure and using nested polymerase chain reaction (PCR) approach to amplify the COX III gene, with primers specific for the parasite mitochondrial DNA.

### Statistical analysis

Continuous data were analyzed as median and/or interquartile range (IQR) or as mean with standard deviation (SD), when normally distributed. Frequencies with percentages were used for categorical data. Overall group differences in the endpoints by patient sub-groups were evaluated using the Kruskal–Wallis test. The Wilcoxon rank-sum rank test was utilized for pair-wise group comparisons. Correlations were expressed as Spearman rank coefficients. *p*-values less than 0.05 were considered statistically significant.

### Ethics

Approval for the study was obtained from the KBTH Institutional Review Board (KBTH—IRB/00010/2020) and the Ghana Health Service Ethics Review Committee (GHS-ERC: 014/02/21). Informed consent was obtained from parents or guardians of all recruited participants, and assent obtained from children ≥age 10 years.

## Results

The median (range) duration of HU treatment (for those on HU) was 28 (3–60) months and the median (range) HU dose was 25 (18–35) mg/kg/day.

### Comparison of RDT-positive SCD vs. steady state SCD (HU+ vs. no-HU)

Selected demographic parameters (age and sex) were comparable between those with acute malaria and those in steady state, whether they were on HU or otherwise. Among those on HU (HU+), participants with acute malaria had significantly higher admission temperature, lower Hb, higher ANC, ALT, and gamma-glutamyl-transferase (GGT) than steady state participants. Among the no-HU group, participants with acute malaria had higher admission temperature, WBC, ANC, RET, total bilirubin (BIL) and urea than participants in steady state (Table 1).

**Table 1:** Baseline (day 0) characteristics of SCD patients (RDT positive) and steady state SCD patients on HU and those not on HU.

Parameter	SCD RDT positive on HU mean ± SD (*n*) median (range) OR *n* (%)	Steady state SCD on HU mean ± SD (*n*) median (range) OR *n* (%)	*p*-value	SCD RDT positive not on HU mean ± SD (*n*) median (range) OR *n* (%)	Steady state SCD not on HU mean ± SD (*n*) median (range) OR *n* (%)	*p*-value	Reference values
Age (years)	7.80 ± 4.02 (10)	7.57 ± 4.18 (58)	0.872	6.77 ± 3.65 (13)	9.06 ± 4.75 (47)	0.112	N/A
	8.00 (2.00 to 14.00)	8.00 (1.00 to 15.00)		5.00 (1.00 to 13.00)	9.00 (0.00 to 20.00)		
Sex			0.742			1.000	N/A
(Female)	5 (50.00%)	23 (39.00%)		6 (46.20%)	23 (50.00%)		
(Male)	5 (50.00%)	36 (61.00%)		7 (53.80%)	23 (50.00%)		
Weight (kg)	23.92 ± 9.87 (10)	26.08 ± 18.73 (57)	0.724	20.51 ± 7.03 (13)	29.65 ± 14.05 (44)	0.028	N/A
	24.05 (8.20 to 37.00)	21.50 (8.20 to 143.00)		19.30 (8.00 to 32.00)	29.00 (7.40 to 64.00)		
Temperature (°C)	38.28 ± 1.42 (10)	36.45 ± 0.43 (58)	<0.001	37.80 ± 1.21 (13)	36.50 ± 0.50 (44)	<0.001	36.5–37.5
	38.35 (36.50 to 40.40)	36.50 (35.60 to 37.90)		37.90 (36.20 to 39.90)	36.50 (35.60 to 37.90)		
Spleen size (cm)	1.20 ± 2.04 (10)	1.00 ± 2.76 (22)	0.839	3.50 ± 4.90 (12)	2.17 ± 2.86 (6)	0.550	N/A
	0.00 (0.00 to 5.00)	0.00 (0.00 to 11.00)		0.75 (0.00 to 13.50)	1.00 (0.00 to 7.00)		
Hemoglobin (g/dL)	6.47 ± 1.27 (9)	8.29 ± 1.34 (52)	<0.001	7.59 ± 2.62 (11)	8.79 ± 1.61 (43)	0.061	8.0–17.0
	5.70 (5.40 to 8.50)	8.20 (5.40 to 11.10)		7.50 (3.80 to 11.50)	8.80 (4.50 to 11.80)		
MCV (fL)	87.51 ± 15.87 (9)	91.69 ± 9.51 (52)	0.280	80.06 ± 13.28 (10)	74.82 ± 9.50 (42)	0.154	86.0–110.0
	95.10 (63.50 to 105.00)	93.20 (73.40 to 122.30)		79.05 (61.20 to 106.10)	73.15 (59.10 to 100.80)		
WBC (10^9^/L)	11.85 ± 4.61 (8)	9.00 ± 4.06 (52)	0.075	15.74 ± 6.22 (11)	10.59 ± 4.70 (43)	0.004	3.0–15.0
	12.46 (6.46 to 18.88)	8.16 (3.47 to 24.76)		15.28 (8.25 to 29.45)	9.55 (4.06 to 26.14)		
Neutrophil number	7.00 ± 4.48 (8)	3.59 ± 2.30 (52)	0.001	7.54 ± 4.69 (10)	4.49 ± 2.43 (43)	0.005	1.50–7.00
	5.47 (2.81 to 15.83)	3.11 (0.36 to 14.91)		5.19 (3.51 to 18.05)	4.08 (1.81 to 14.11)		
Lymphocyte number	4.82 ± 3.19 (9)	4.27 ± 1.93 (52)	0.482	6.63 ± 4.11 (9)	4.78 ± 2.42 (43)	0.073	1.00–3.70
	4.21 (1.79 to 12.24)	4.12 (1.57 to 10.66)		5.89 (2.32 to 15.13)	4.40 (1.69 to 12.05)		
Platelets (10^9^/L)	241.22 ± 114.80 (9)	309.46 ± 154.66 (52)	0.212	235.18 ± 125.71 (11)	319.07 ± 144.99 (43)	0.085	50–400
	243.00 (94.00 to 415.00)	324.00 (18.80 to 678.00)		205.00 (96.00 to 489.00)	270.00 (42.00 to 678.00)		
Reticulocyte number	0.21 ± 0.06 (8)	0.21 ± 0.08 (52)	0.862	0.27 ± 0.22 (9)	0.18 ± 0.10 (43)	0.049	0.0000–0.9999
	0.22 (0.09 to 0.29)	0.19 (0.08 to 0.41)		0.19 (0.12 to 0.82)	0.18 (0.02 to 0.44)		
Total bilirubin (μmol/L)	34.42 ± 27.50 (8)	37.46 ± 19.79 (55)	0.701	62.87 ± 55.94 (10)	32.56 ± 15.19 (43)	0.003	1.71–20.52
	24.09 (18.33 to 101.66)	30.76 (10.91 to 105.60)		34.22 (14.37 to 172.52)	28.74 (11.97 to 68.99)		
AST (U/L)	63.12 ± 34.43 (8)	57.29 ± 33.63 (55)	0.649	50.30 ± 18.54 (10)	44.14 ± 19.46 (43)	0.368	0–31
	65.00 (22.00 to 115.00)	53.00 (23.00 to 258.00)		48.00 (25.00 to 82.00)	38.00 (18.00 to 94.00)		
ALT (U/L)	47.38 ± 26.37 (8)	27.35 ± 20.51 (55)	0.016	28.70 ± 11.26 (10)	25.28 ± 12.72 (43)	0.438	0–34
	59.50 (11.00 to 85.00)	23.00 (12.00 to 157.00)		29.00 (9.00 to 45.00)	20.00 (9.00 to 59.00)		
GGT (U/L)	52.25 ± 46.91 (8)	26.33 ± 16.90 (55)	0.003	66.90 ± 94.06 (10)	32.40 ± 33.32 (42)	0.056	9–39
	32.00 (13.00 to 125.00)	23.00 (8.00 to 106.00)		24.50 (17.00 to 319.00)	25.00 (12.00 to 232.00)		
Na (mmol/L)	142.28 ± 6.64 (8)	142.22 ± 8.24 (55)	0.987	141.12 ± 6.51 (10)	138.12 ± 8.10 (44)	0.280	135–145
	142.70 (130.30 to 153.70)	144.40 (126.00 to 155.70)		142.30 (127.40 to 151.60)	137.45 (115.00 to 151.50)		
K (mmol/L)	5.36 ± 2.78 (8)	6.83 ± 2.34 (55)	0.110	5.89 ± 1.68 (10)	5.61 ± 1.90 (44)	0.666	3.5–5.5
	4.32 (3.08 to 11.41)	6.15 (3.49 to 15.14)		6.03 (4.01 to 8.96)	5.69 (0.13 to 12.47)		
Urea (mmol/L)	2.95 ± 1.45 (8)	2.19 ± 0.97 (54)	0.057	4.60 ± 4.27 (10)	3.04 ± 1.40 (43)	0.048	1.7–8.3
	2.55 (1.40 to 5.20)	1.85 (0.40 to 4.90)		2.90 (1.90 to 16.00)	2.70 (1.30 to 7.20)		
Creatinine (μmol/L)	44.98 ± 11.07 (8)	48.21 ± 12.57 (53)	0.496	42.09 ± 19.03 (10)	48.16 ± 13.45 (43)	0.241	44.2–88.4
	39.52 (33.90 to 63.47)	47.47 (6.33 to 75.06)		39.57 (6.12 to 64.71)	49.81 (17.41 to 92.57)		

MCV, mean corpuscular volume; WBC, white blood cells; AST, aspartate aminotransferase; ALT, alanine aminotransferase; GGT, gamma-glutamyl-transferase; Na, sodium; K, potassium; N/A, non-applicable.

### RDT-positive SCD HU+ versus RDT-positive SCD no-HU: baseline clinical signs

The HU+ participants (*n* = 10) were all HbSS genotype, while the no-HU participants comprised HbSS (*n* = 9) and HbSC (*n* = 4) genotypes. Cardiac murmurs and hepatomegaly were observed more frequently in the HU+ group on day 0, while jaundice and splenomegaly were more prevalent in the no-HU group (Supplementary Table S1). Aside from a (statistically non-significant) larger spleen size in the no-HU group, there were no other differences between the two groups on day 0.

### RDT-positive SCD HU+ versus RDT-positive SCD no-HU: parasitaemia carriage and clearance

**Figure 1:**
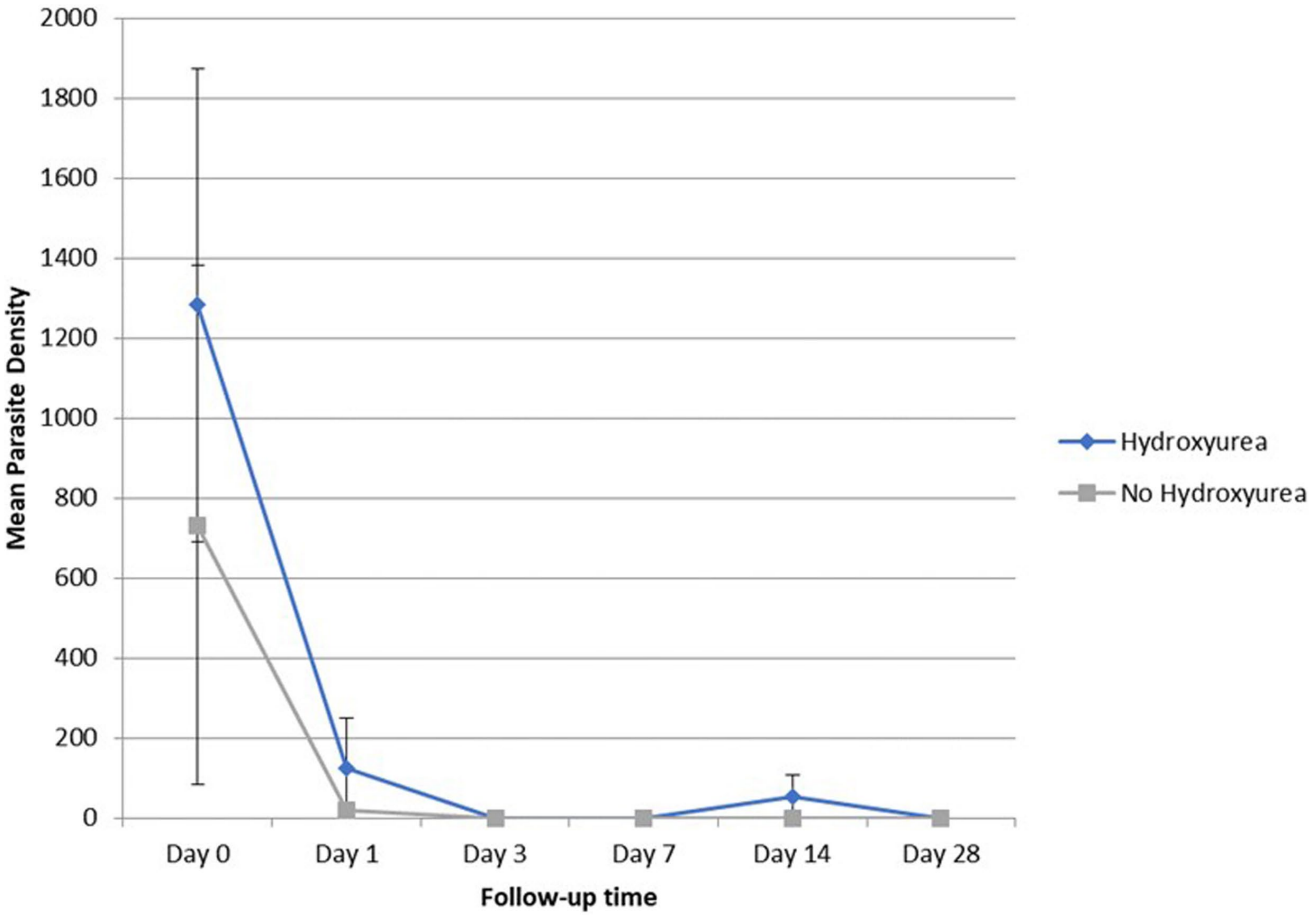
Geometric mean (±SEM) parasite density of SCD (HU versus no HU) patients from day 0 to day 28.

Overall, parasitaemia was detected by microscopy among 30.4% (7/23) of the RDT-positive SCD participants (4/10 HU+ and 3/13 no-HU; *p* = 0.38), and by PCR among 60.9% (8/10 HU+ and 6/13 no-HU) (Supplementary Table S2). Geometric mean *Plasmodium falciparum* parasite density was higher (though non-significantly), in the HU+ group (Table 2). None of the study participants had (microscopic) detectable parasitaemia by day 3, or on any of the subsequent follow up days, except one study participant: (14 years-old male, HbSS, who had a surgical splenectomy the previous year) who was seen with recurrent parasitaemia on day 14. There was no significant difference in parasite clearance between the HU+ and no-HU groups (Figure 1).

**Table 2:** Baseline (day 0) characteristics of RDT positive SCD patients (HU+ versus no-HU).

Parameter	SCD RDT positive on HU mean ± SD (*n*) median (range) OR *n* (%)	SCD RDT positive not on HU mean ± SD (*n*) median (range) OR *n* (%)	*p*-value	Reference values
Age (years)	7.80 ± 4.02 (10)	6.77 ± 3.65 (13)	0.528	N/A
	8.00 (2.00 to 14.00)	5.00 (1.00 to 13.00)		
Sex			1.000	N/A
(Female)	5 (50.00%)	6 (46.20%)		
(Male)	5 (50.00%)	7 (53.80%)		
Weight (kg)	23.92 ± 9.87 (10)	20.51 ± 7.03 (13)	0.343	N/A
	24.05 (8.20 to 37.00)	19.30 (8.00 to 32.00)		
Temperature (°C)	38.28 ± 1.42 (10)	37.80 ± 1.21 (13)	0.392	36.5–37.5
	38.35 (36.50 to 40.40)	37.90 (36.20 to 39.90)		
Geometric mean (95% CI) parasite density (/μL)	2930.3 (1325.9–6476.2)	1,060 (7.53–149, 432.5)	0.74	N/A
Spleen size (cm)	1.20 ± 2.04 (10)	3.50 ± 4.90 (12)	0.182	N/A
	0.00 (0.00 to 5.00)	0.75 (0.00 to 13.50)		
Hemoglobin (g/dL)	6.47 ± 1.27 (9)	7.59 ± 2.62 (11)	0.256	8.0–17.0
	5.70 (5.40 to 8.50)	7.50 (3.80 to 11.50)		
MCV (fL)	87.51 ± 15.87 (9)	80.06 ± 13.28 (10)	0.281	86.0–110.0
	95.10 (63.50 to 105.00)	79.05 (61.20 to 106.10)		
WBC (10^9^/L)	11.85 ± 4.61 (8)	15.74 ± 6.22 (11)	0.154	3.0–15.0
	12.46 (6.46 to 18.88)	15.28 (8.25 to 29.45)		
Neutrophil number	7.00 ± 4.48 (8)	7.54 ± 4.69 (10)	0.809	1.50–7.00
	5.47 (2.81 to 15.83)	5.19 (3.51 to 18.05)		
Lymphocyte number	4.82 ± 3.19 (9)	6.63 ± 4.11 (9)	0.311	1.00–3.70
	4.21 (1.79 to 12.24)	5.89 (2.32 to 15.13)		
Platelets (10^9^/L)	241.22 ± 114.80 (9)	235.18 ± 125.71 (11)	0.913	50–400
	243.00 (94.00 to 415.00)	205.00 (96.00 to 489.00)		
Reticulocyte number	0.21 ± 0.06 (8)	0.27 ± 0.22 (9)	0.471	0.0000–0.9999
	0.22 (0.09 to 0.29)	0.19 (0.12 to 0.82)		
Total bilirubin (μmol/L)	34.42 ± 27.50 (8)	62.87 ± 55.94 (10)	0.208	1.71–20.52
	24.09 (18.33 to 101.66)	34.22 (14.37 to 172.52)		
AST (U/L)	63.12 ± 34.43 (8)	50.30 ± 18.54 (10)	0.326	0–31
	65.00 (22.00 to 115.00)	48.00 (25.00 to 82.00)		
ALT (U/L)	47.38 ± 26.37 (8)	28.70 ± 11.26 (10)	0.059	0–34
	59.50 (11.00 to 85.00)	29.00 (9.00 to 45.00)		
GGT (U/L)	52.25 ± 46.91 (8)	66.90 ± 94.06 (10)	0.694	9–39
	32.00 (13.00 to 125.00)	24.50 (17.00 to 319.00)		
Na (mmol/L)	142.28 ± 6.64 (8)	141.12 ± 6.51 (10)	0.716	135–145
	142.70 (130.30 to 153.70)	142.30 (127.40 to 151.60)		
K (mmol/L)	5.36 ± 2.78 (8)	5.89 ± 1.68 (10)	0.620	3.5–5.5
	4.32 (3.08 to 11.41)	6.03 (4.01 to 8.96)		
Urea (mmol/L)	2.95 ± 1.45 (8)	4.60 ± 4.27 (10)	0.314	1.7–8.3
	2.55 (1.40 to 5.20)	2.90 (1.90 to 16.00)		
Creatinine (μmol/L)	44.98 ± 11.07 (8)	42.09 ± 19.03 (10)	0.708	44.2–88.4
	39.52 (33.90 to 63.47)	39.57 (6.12 to 64.71)		

MCV, mean corpuscular volume; WBC, white blood cells; AST, aspartate aminotransferase; ALT, alanine aminotransferase; GGT, gamma-glutamyl-transferase; Na, sodium; K, potassium; N/A, non-applicable.

### RDT-positive SCD HU+ versus RDT-positive SCD no-HU: trends in haematological parameters

**Figure 2:**
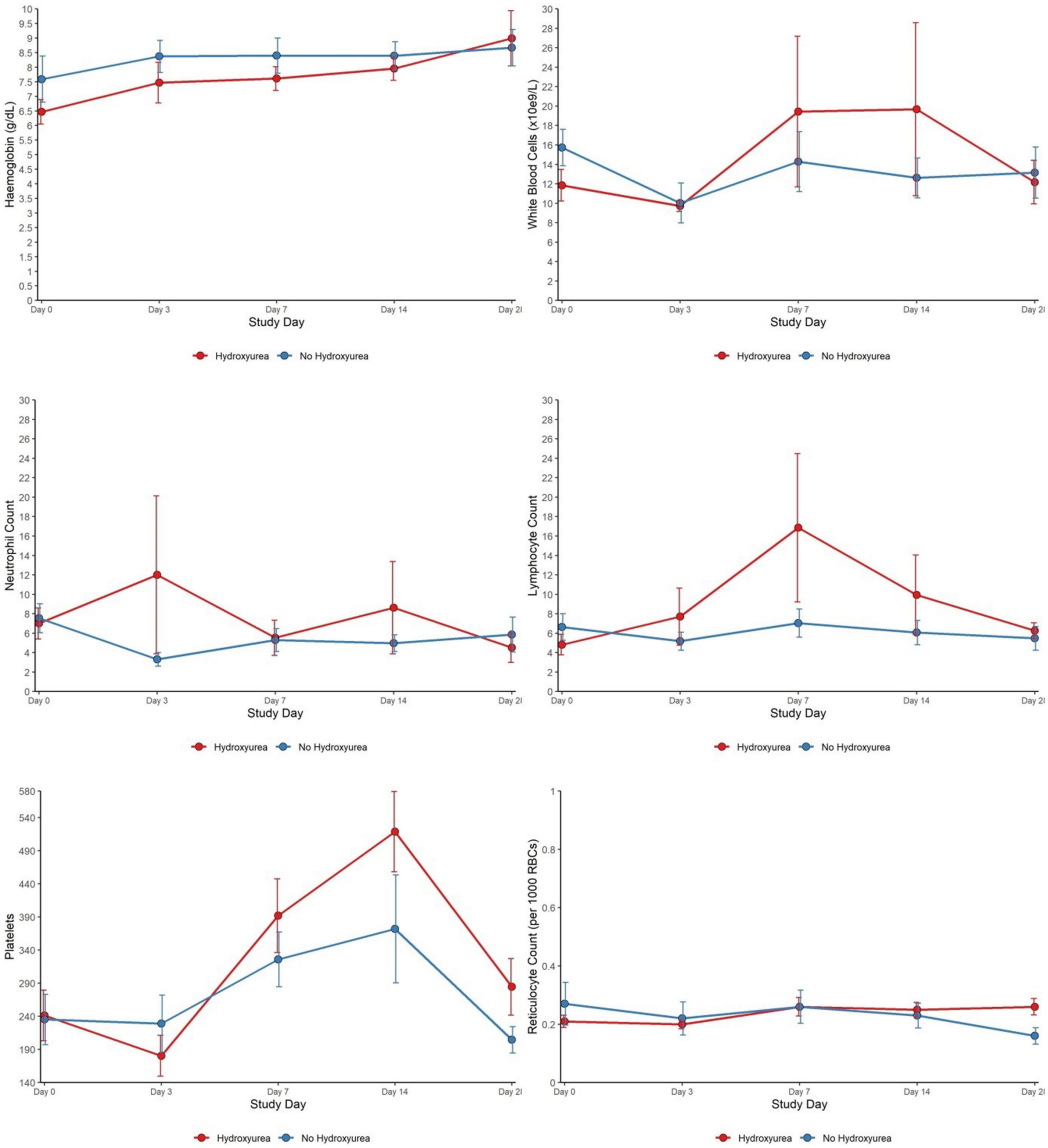
Haematological characteristics of SCD patients (HU versus no HU) who were RDT positive and received AL from day 0 to day 28.

Total WBC and ANC were lower in the HU+ group on days 0 and 28 but the pattern was reversed between days 3 through to 14. Platelet counts, while comparable between the HU+ and no-HU groups on day 0, declined more sharply in the HU+ group by day 3, subsequently reversing its pattern with a peak on day 14 and comparatively higher levels by day 28. Hb concentration was initially comparatively lower in the HU+ group (day 0) but reverted to slightly higher levels by day 28. The change in lymphocyte count (day 0 and day 7) between HU+ (mean change; 95% CI) [2.74 (−5.38 to 58.57)] and no-HU [−0.34 (−3.19 to 4.44)] groups was significant (*p* = 0.024). Similarly, the change in RET count between HU+ [0.24 (0.17 to 0.37)] and no-HU [0.15 (0.09 to 0.27)] was significant on day 28 (*p* = 0.022). Graphical presentation of trends in haematological parameters from day 0 to day 28 are shown (Figure 2).

### RDT-positive SCD HU+ versus RDT-positive SCD no-HU: trends in clinical chemistry parameters

**Figure 3:**
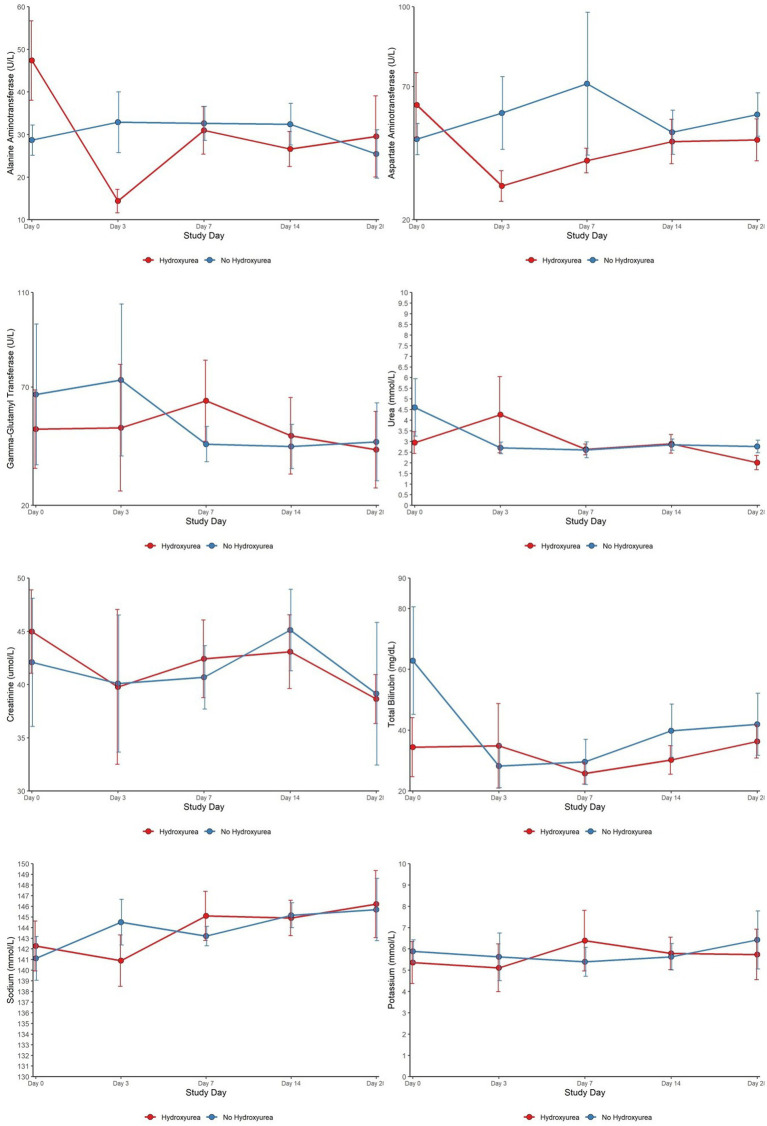
Clinical chemistry characteristics of SCD patients (HU versus no HU) who were RDT positive and received AL from day 0 to day 28.

Day 0 (pre-treatment) higher ALT and AST levels in the HU+ group declined sharply, attaining a nadir on day 3. Comparatively, lower day 0 GGT and urea levels in the HU+ group trended upwards between days 3 and 14, reverting to comparable levels as the no-HU group, by day 28. Between days 0 and 3, mean (95% CI) changes in BIL [HU+ −4.44 (−16.36 to 20.74)]; no-HU [−18.37 (−108.79 to −7.16)]; and ALT HU+ [−4.00 (−48.55 to 6.00)], no-HU [7.00 (−22.00 to 22.00)], were significant (*p* = 0.018 for both parameters). ALT changes during subsequent follow up days: days 0 and 7 (*p* = 0.010) and days 0 and 14 (*p* = 0.023), were also significant. The patterns of changes in other clinical chemistry parameters from day 0 to day 28 are shown (Figure 3).

### RDT-positive SCD HU+ versus RDT-positive SCD no-HU: adverse event occurrence on day 28

Using the CTCAE criteria, a cumulative total of 13.9% (11/79) adverse events occurred in the HU+ group, compared with 14.3% (10/70) in the no-HU group, by day 28 (*p* = 0.94). Elevated bilirubin (*n* = 5; grade one = 1; grade two = 4 in either group) was the most common adverse event (Supplementary Table S3).

## Discussion

Malaria and SCD both remain diseases of public health significance. Deployment of HU as disease modifying therapy for SCD in malaria endemic countries has the potential to improve SCD care; however, concomitant antimalarial drug administration within the context of HU therapy may require further safety data and or assurance as well as demonstration of efficacy. This study provides preliminary data on systematic changes in haematological and clinical chemistry parameters and preliminary efficacy data among SCD patients with uncomplicated malaria on HU therapy treated with AL. The study also provides data on the dynamics of these changes, from acute malaria illness through recovery, between antimalarial treated HU+ and no-HU recipients. Additionally, the baseline data of the malaria treated SCD HU+ and no-HU participants have been compared with the corresponding steady state SCD HU+ or no-HU group. The findings thus provide data on malaria- or SCD-distinctive changes during the acute and immediate post-recovery periods.

Our results showed lower Hb levels in participants with acute malaria compared to those in steady state, although this was only significant in the HU+ group. While RET was similar in those on HU (RDT-positive vs. steady state), it was significantly higher in the RDT-positive no-HU group compared to steady state no-HU. SCD causes alterations in blood cell indices characterized by a reduction in Hb and increase in RET, while malaria also causes accelerated breakdown of both parasitized and uninfected red cells, potentially worsening the effect on Hb and RET (21, 22). Although HU is known to cause reticulocytopenia, the expected increase in RET in the context of acute malaria haemolysis was not initially seen in the RDT-positive HU+ group, despite having significantly lower Hb than those in steady state. Over the follow up period, the RET was similar in both RDT-positive HU+ and no-HU groups until day 28, when the HU+ group had significantly higher RET in keeping with greater bone marrow stimulation in this sub-group as their Hb levels were also higher by day 28 (23). Further evaluation with a larger group of participants is needed to verify these findings.

WBC, ANC, and platelets are elevated in SCD steady state, due to underlying chronic inflammation and increased expression of endothelial adhesion molecules. The myelosuppressive effect of HU usually counteracts these changes (8, 24). We however found significantly higher ANC levels in those with acute malaria vs. steady state, irrespective of HU status. In the REACH study, HU-induced myelosuppression with ANC <3.0, was protective against malaria through incompletely defined mechanisms that may include decreased inflammation (10). In the current study, WBC was also increased in both HU+ and no-HU malaria groups compared to those in steady state, although this was not statistically significant for the HU+ group. Surprisingly, platelet counts were comparable across all groups at baseline. In malaria pathogenesis, platelets play both beneficial and deleterious roles, with malaria being a known cause of thrombocytopenia. Thrombocytopenia is also a marker of disease severity in *P. falciparum* and *P. vivax* infections (25, 26). It is not clear why our acute malaria cohorts did not show this expected platelet drop, but the lack of differences compared to those in steady state could be because they had uncomplicated infection. Interestingly, comparing only the HU+ malaria and no-HU malaria groups, we found marked differences between groups in the platelet counts over the 28 days follow up period, although the patterns of change were similar, with both groups showing relatively lower platelet counts on day 3, a peak on day 14 and values close to baseline on day 28.

Although the HU+ malaria group had a higher mean parasite density than the no-HU malaria group, this was not statistically significant. SCD does not share the protective effect of HbS against severe *P. falciparum* malaria seen in sickle cell trait, yet parasite burden is usually lower, and SCD individuals develop clinical malaria at lower parasitaemia than non-SCD. HbS-containing red cells may present a hostile environment for malaria parasite development as infected sickled red cells are preferentially removed from circulation by splenic macrophages. HU reduces HbS concentration and increases HbF levels in red cells, and improvements in deformability and cellular hydration, leading to red cells less prone to premature haemolysis. Increased HbF may also be associated with restriction of parasite growth *in vitro* (27), and HU may act synergistically with antimalarials to prevent parasite growth (28). *In vitro* studies show that ribonucleotide reductase inhibitors interfere with malaria parasite development at the schizont stage through inhibition of DNA synthesis, while HU may increase expression of endothelial intracellular adhesion molecule 1, facilitating parasite adhesion to the endothelium (29).

The finding of higher mean parasitaemia at enrolment in the HU+ malaria group could have caused a significant drop in haemoglobin at the start of infection and may account for the (unexpectedly) similar haemoglobin levels at malaria diagnosis in the HU+ and no-HU groups. It may also explain the higher prevalence of anaemia-associated clinical signs at presentation in the HU+ group. The increased Hb by days 7 and 28, in both groups, are in keeping with complete recovery from the acute malaria infection.

There were comparable ANC between the HU+ and no-HU malaria groups at baseline and significantly higher ANC in the HU+ group on days 3 and 14 but a reverse trend by day 28. However, the days 3 through 14 ANC among the HU+ group was skewed by the single post-splenectomy participant who had persistent parasitaemia through day 14. Neutrophils play a crucial role in the body’s defense against pathogens and are implicated in the phagocytosis of malaria parasite-infested RBC and free merozoites. Neutrophils also produce reactive oxygen species that inhibit parasite growth (30). While these changes may be incorrectly interpreted as evidence of underlying bacterial infection, the degree of neutrophilia is linked to an increased likelihood of developing severe malaria due to the formation of neutrophil extracellular traps and transient immunosuppression (30), and both leukocytosis and neutrophilia represent adverse prognostic factors for severe SCD-related complications such as stroke. The finding of persistent parasitaemia-associated neutrophilia signals and confirms the adverse effects of malaria among SCD patients and the need for prompt malaria treatment in this patient population.

Lymphocyte counts on days 3 and 28 were comparable between the HU+ and no-HU groups. However, the corresponding counts were markedly different between days 7 and 14. While the implication of these dynamics are unknown, T cell lymphocytes play a role in priming phagocytic cells to capture or kill malaria parasites as well as helping B lymphocytes produce functional anti-parasitic antibodies (31). Lymphocytes are also important in SCD pathophysiology as invariant natural killer T cells have been implicated as a critical immune profile that potentiates SCD and may act as a possible therapeutic target. The implication of the comparatively higher lymphocyte counts in the HU+ group in the immediate post-treatment phase are unclear, even as HU is known to have antiproliferative effects on T lymphocytes *in vitro* (32) and specifically targets quiescent lymphocytes *in vivo* (33).

Despite lack of any significant differences in biochemical parameters on Day 0 between both malaria groups, the temporal patterns showed marked variations in AST and ALT between days 0 and 28. The nadir of ALT and AST occurred in the HU+ group on day 3, with no change in BIL on day 3—a finding that was not seen in the no-HU group. The latter group had an increase in the AST and ALT values on day 3, consistent with previous reports of self-limiting, disease and/or drug-related liver injury (17, 34). Although HU can cause acute elevations in liver enzymes, it is well tolerated in children with SCD with no short- or long-term hepatotoxicity. In a single treatment centre in the United Kingdom, significant reductions in the ALT and AST were seen in children with sickle cell anaemia who had good adherence to HU, signifying a beneficial effect on hepatocytes (35).

Being the most prescribed ACT in sub-Saharan Africa (36), several systematic studies have shown an overall high (>95%) AL treatment success in sub-Saharan Africa (37, 38), with comparatively high efficacy rates reported among SCD patients (12, 39). However, a lower AL therapeutic efficacy has been reported among young underweight children from Africa (40), while AL treatment has also been associated with a higher treatment failure risk, or reduced lumefantrine absorption in underweight children (41, 42). Among the general population of children in Burkina Faso and Uganda, there have also been recent concerns of AL treatment failure (43, 44). Furthermore, AL treatment has been associated with delayed parasite clearance among SCD patients and SCD-associated pathophysiological changes such as impairment of hepatorenal function, albuminuria (45), malnutrition (46), or alterations in blood cell indices, may act in concert, with implications for overall treatment outcome in SCD patients. In this study, however, absent parasitaemia in all study participants by day 3 demonstrates rapid parasite clearance and supports the continued high AL efficacy in our study population. The recurrence of parasitaemia in a splenectomized patient, is consistent with evidence of prolonged parasite clearance in individuals with impaired splenic function and with the findings from a recent report from Sudan, of parasite recrudescence after antimalarial treatment in a splenectomized SCD patient on HU (47).

## Conclusion

Children with SCD treated with AL for acute uncomplicated malaria had rapid parasite clearance and high day 28 cure rates irrespective of whether they were on prior HU therapy. However, distinct pattern of changes in haematological and blood chemistry parameters between HU+ and no-HU groups in this pilot study, including rapid decline in ALT and AST among the HU+, in contrast with elevated ALT and BIL levels in the no-HU group during the acute illness phase, suggest that further focused studies are needed to evaluate the significance of these observations in the specific context of long term safety of HU during acute malaria and its treatment. Furthermore, our finding of recurrent parasitaemia in a single splenectomized patient, highlights the need for studies to refine the role of the spleen in SCD patients with acute malaria and to advocate for antimalarial preventive interventions for high-risk SCD patients in endemic areas.

### Limitations

Although the 28 days follow up schedule was consistent with WHO recommendations for antimalarial efficacy studies, a longer follow up would be a more optimal design in terms of determining the natural history and dynamics of changes in haematological or clinical chemistry parameters and long term safety implications. A heterogeneous SCD population with different phenotypes would be a more robust approach to unravelling the clinical and para-clinical effects of HU in this population.

## Data availability statement

The raw data supporting the conclusions of this article will be made available by the authors, without undue reservation.

## Ethics statement

The studies involving humans were approved by Institutional Review Board, Korle Bu Teaching Hospital; and the Ghana Health Service Ethics Review Committee. The studies were conducted in accordance with the local legislation and institutional requirements. Written informed consent for participation in this study was provided by the participants’ legal guardians/next of kin.

## Author contributions

CS: Conceptualization, Data curation, Investigation, Methodology, Supervision, Validation, Writing – original draft, Writing – review & editing. SKA: Conceptualization, Data curation, Investigation, Methodology, Supervision, Validation, Writing – original draft, Writing – review & editing. AA: Data curation, Investigation, Methodology, Supervision, Validation, Writing – original draft, Writing – review & editing. MN: Conceptualization, Investigation, Methodology, Supervision, Validation, Writing – review & editing. YB: Conceptualization, Investigation, Methodology, Supervision, Validation, Writing – review & editing. NS: Conceptualization, Investigation, Methodology, Supervision, Validation, Writing – review & editing. SYA: Investigation, Methodology, Supervision, Validation, Writing – review & editing. AS: Data curation, Formal analysis, Investigation, Methodology, Validation, Writing – review & editing. LH: Conceptualization, Funding acquisition, Methodology, Project administration, Resources, Supervision, Validation, Writing – review & editing. MO: Conceptualization, Funding acquisition, Investigation, Methodology, Project administration, Resources, Supervision, Validation, Writing – review & editing. GA: Conceptualization, Data curation, Formal analysis, Funding acquisition, Investigation, Methodology, Project administration, Resources, Supervision, Validation, Visualization, Writing – original draft, Writing – review & editing.
